# Genomics of adaptation and acclimation: from field to lab and back

**DOI:** 10.1093/nsr/nwz173

**Published:** 2019-11-13

**Authors:** Scott V Edwards

**Affiliations:** 1 Department of Organismic and Evolutionary Biology and Museum of Comparative Zoology, Harvard University, USA; 2 Reviewer of NSR

These days, the sequencing of genomes and discovery of genomic regions associated with adaptive differences between populations and species are commonplace. What is less well understood is the role of gene expression in the ability of species to invade novel habitats and survive environmental challenges [1]. Such an understanding is important, especially as numerous species are introduced to new regions of the globe through human intervention and spread as invasive species, often leaving ecological chaos in their wake. Additionally, we are only just beginning to understand the role of gene expression in acclimation of species to novel habitats [2,3]. Both adaptation and acclimation are important in the ability of species to survive and invade new habitats [4,5]. Measuring the relative contribution of these factors to invasive ability, and identifying signals of their genomic underpinnings, is a major task of evolutionary biology.

Transplantation experiments involving the movement of live animals from one field site to another are commonly employed to understand the changes species undergo when they acclimatize to a new environment. Such experiments are commonplace in plants, occasionally are undertaken in vertebrate animals [3,6] and invariably reveal a host of phenotypic changes in the new environment, including changes in gene expression [3]. When wholly field-based transplantation experiments are not possible, transplanting individuals to a simulated field environment is a good alternative. Such an experiment, along with copious genomic and transcriptomic surveys, was undertaken by Qu *et al.* in this issue [7]. The strength of their study—one of the few studies in birds to assay gene expression in the wild—lies in their ability to compare transcriptional and phenotypic responses of birds in the wild at both high- and low-elevation sites. Using demographic modeling, they first ruled out the possibility of population structure causing genome-wide differentiation of allele frequencies—and possibly gene expression—in their study populations. They found little structure, because the sparrows they studied likely invaded the Tibetan Plateau very recently, with the permanent establishment of humans and agriculture a few thousand years ago. Then, Qu *et al.* studied changes to muscle morphology in birds transported from the lowland sites to hypoxia-simulated laboratory conditions. They did not find significant changes in muscle morphology (capillary number or fiber area) in the acclimated sparrows after 30 days. Given the large number of gene-expression differences they found between natural lowland and highland sparrows, and the several regulatory pathways with respiratory function that they identified, it would have been interesting to have examined gene expression in the laboratory-acclimated sparrow population [5]. One can imagine that it may be easier and faster for organisms to acclimate via gene-expression changes than by morphological changes. This experiment would be an excellent follow-up to further connect the laboratory and field discoveries.

Overall, Qu *et al.* have identified a very promising species, and an even more promising environmental setting in the Tibetan Plateau [8,9], to investigate the genomics of adaptation and acclimation. Their study, combining genomics, transcriptomics, muscle morphology and physiology, exemplifies the modern meaning of ‘integrative biology’.

***Conflict of interest statement***. None declared.
